# The New Ion-Selective Electrodes Developed for Ferric Cations Determination, Modified with Synthesized Al and Fe−Based Nanoparticles

**DOI:** 10.3390/s22010297

**Published:** 2021-12-31

**Authors:** Andrea Paut, Ante Prkić, Ivana Mitar, Lucija Guć, Marijan Marciuš, Martina Vrankić, Stjepko Krehula, Lara Tomaško

**Affiliations:** 1 Faculty of Chemistry and Technology, University of Split, Ruđera Boškovića 35, 21000 Split, Croatia; andrea.paut@ktf-split.hr (A.P.); lguc@pmfst.hr (L.G.); laratomasko6@gmail.com (L.T.); 2 Faculty of Science, University of Split, Ruđera Boškovića 33, 21000 Split, Croatia; imitar@pmfst.hr; 3 Division of Materials Chemistry, Ruđer Bošković Institute, Bijenička cesta 54, 10000 Zagreb, Croatia; marijan.marcius@irb.hr (M.M.); krehul@irb.hr (S.K.); 4 Division of Materials Physics and Center of Excellence for Advanced Materials and Sensing Devices, Ruđer Bošković Institute, Bijenička cesta 54, 10000 Zagreb, Croatia; martina.vrankic@irb.hr

**Keywords:** potentiometry, nanoparticles, microwave synthesis, iron(III) cations

## Abstract

The solid-state ion-selective electrodes presented here are based on the FePO_4_:Ag_2_S:polytetrafluoroethylene (PTFE) = 1:1:2 with an addition of (0.25–1)% microwave-synthesized hematite (α-Fe_2_O_3_), magnetite (Fe_3_O_4_), boehmite [γ-AlO(OH)], and alumina (Al_2_O_3_) nanoparticles (NPs) in order to establish ideal membrane composition for iron(III) cations determination. Synthesized NPs are characterized with Fourier-Transform Infrared (FTIR) spectroscopy, Powder X-Ray Diffraction (PXRD), and Scanning Electron Microscopy (SEM) with Energy Dispersive Spectroscopy (EDS). The iron oxides NPs, more specifically, magnetite and hematite, showed a more positive effect on the sensing properties than boehmite and alumina NPs. The hematite NPs had the most significant effect on the linear range for the determination of ferric cations. The membrane containing 0.25% hematite NPs showed a slope of −19.75 mV per decade in the linear range from 1.2∙10^−6^ to 10^−2^ mol L^−1^, with a correlation factor of 0.9925. The recoveries for the determination of ferric cations in standard solutions were 99.4, 106.7, 93.6, and 101.1% for different concentrations.

## 1. Introduction

The electroanalytical methods, with emphasis on ion-selective electrodes, are one of the most rapidly developing detection methods due to the wide range of applications and meet the requirements of sensitivity, selectivity, small size, ease of use, portability, timeliness, and low cost. Ion-selective electrodes can determine the exact concentration of the analyte over a wide concentration range, allowing the use of a single method to determine the analyte in a variety range of samples without pretreatment of the sample matrix [1,2]. Nanotechnology, i.e., the synthesis and usage of nanomaterials such as nanotubes, nanowires, nanofibers, nanorods, NPs, nanocomposites, and other nanostructures, has recently emerged as one of the most exciting and rapidly developing areas of analytical and electroanalytical chemistry. Various nanomaterials, especially NPs with different properties, have found a wide application in many types of electrochemical sensors [3,4]. An application of nanomaterials in chemosensors and biosensors is based on their specific properties, especially the large surface-to-volume ratio, which favors stronger interaction with analytes when nanostructures are part of the surface layer, as well as their good conductivity, electrocatalytic activity, and high mechanical strength [5]. The performance of sensor modification with NPs includes: (i) nanomaterials as solid contacts in solid-state ion-selective electrodes, (ii) nanomaterials (or ionophore-modified nanomaterials) dispersed in ion-selective electrodes, and (iii) nanomaterial-based biosensors [4]. The metal oxide NPs are widely used as sensor and biosensor modifications thanks to their catalytic and conductive properties and their ability to roughen the conductive sensor interface. This particular area of scientific research is constantly evolving [6,7]. Some of the properties of these NPs depend mainly on their size, which can be controlled by different synthesis methods, at the physical or chemical level [8]. Namely, numerous metal oxide NPs, i.e., manganese [9,10], titanium [11], zinc [12,13,14], cobalt [15], nickel [16], and iron oxides [13] have been used in electrode modification processes.

Due to their biocompatibility and non-toxicity, the iron oxide NPs occupy a special place in improving the properties of electrochemical sensors. Moreover, these NPs are easy to produce and offer a wide range of biomedical applications, especially hematite and magnetite [1,6]. The iron oxide NPs are used as sensor modifiers for the determination of various analytes, such as hydrogen peroxide [17], glucose [18], Pb, Zn, Cd [19], Cl [13], F [20], nitrites [21] and some organic compounds [22,23,24]. The technology of iron oxide NPs synthesis has been highly developed and brought to a level where it is possible to obtain the desired phase and size of NPs by defining the synthesis process [25].

Although boehmite and alumina NPs are not so commonly used as electrochemical modifiers, the nanoporous electrochemical sensors based on alumina membranes have been recently used as biosensors. These types of modified sensors are used to detect viruses, proteins, and pathogens with exceptional sensitivity [26].

Iron is recognized as an essential nutritional element for all life forms. It is found as a cofactor in various enzymes and is very important for oxygen transport and electron transfer in the human body. Although the daily requirement of iron for humans is set at 8–18 mg, iron has been found to be toxic in an excessive concentration due to its pro-oxidant activity. Accordingly, it can be concluded that iron can be both essential and toxic to human health depending on the concentration [27]. Due to these facts, it is extremely important to develop a new, simple, relatively fast, inexpensive, and reliable method for the determination of iron content in food products, beverages, and food supplements.

Among different types of potentiometric sensors used for ferric cations determination, most of them are based on conductive polymers [28,29,30,31,32,33,34,35], carbon materials electrodes [36,37], and to a small extent with those based on iron salts [38]. Silver sulfide was used as a conductor in Ref. [38], where ferric cations were incorporated in membrane composition, unlike the Ref. [39] where Ag_2_S-CuS mixture was used.

In this work, we investigate which type of NPs is the best suited to improve the detection properties of the ion-selective electrode for iron(III) cations. The miniaturized electrode based on the ferric phosphate (FePO_4_), Ag_2_S, and PTFE described in previous work [40] was modified with the hematite, magnetite, boehmite, and alumina oxide NPs, and their influence on the detection limit and sensitivity of the electrode was studied. Ferric-selective electrodes constructed from sparingly soluble salts that had been reported before showed linearity (1∙10^−5^–1∙10^−2^) mol L^−1^ [38,39] with a limit of detection of 5.1∙10^−6^ mol L^−1^ [38]. The synthesis process of hematite NPs used for electrode modification is described in detail in [25], while the synthesis of magnetite, boehmite, and alumina NPs is presented below. This work, with a detailed description of synthesized NPs as modifications for the ion-selective electrodes, is a great step forward compared to previous research.

Ion-selective electrodes are often modified with different types of metal nanomaterials, which is a combination of the simplicity of the potentiometric technique with the improvement of the sensor properties by their modernization with nanostructured materials, thus combining the advantages of these two scientific fields, which is the object of our research and thus, this paper.

## 2. Materials and Methods

### 2.1. Al and Fe-Based NPs Synthesis

All solutions used for the NPs synthesis were prepared by dissolving a precisely weighed mass of a required solid chemical in ultrapure water that was prepared using the Millipore Simplicity 185 purification system (Millipore, Burlington, MA, USA) with a resistivity of 18.2 MΩ cm^−1^ at 25 °C. Mixtures of the corresponding chemicals were prepared at room temperature (RT) in plastic cuvettes and, after mixing, transferred to a non-stirred Milestone Teflon-lined pressure vessel (vessel volume up to 100 mL; maximum pressure of 100 bar and temperature of 300 °C). The samples were heated in a microwave oven (Milestone flexiWave SK15, Milestone, S.r.l., Sorisole, Italy) equipped with the ATC 400 sensor. The ATC sensor allows direct temperature control via microwave transparent fiber optic sensor up to 300 °C; magnetron frequency 2450 MHz; magnetron power 2 × 950 Watt; power supply 230 V, 50–60 Hz,) at the prevailing temperature according to a microwave oven program with the rotor turned on and continuous microwave emission at 800 W. After the reaction time, the obtained precipitates were centrifuged (Beckman Avanti J-25, Indianapolis, IN, USA) and washed several times with ultrapure water and ethanol. After the first centrifugation cycle, the mother liquor was isolated, and the pH of the solution was measured using a pH meter (MP220 Basic Mettler Toledo, Columbus, OH, USA). All precipitates were dried in a vacuum dryer (Thermo Scientific, 3608–1CE, Waltham, MA, USA) at temperatures that are listed in Table 1. In the case of alumina NPs, the precipitates obtained after the microwave synthesis and vacuum drying were calcined in a furnace (Vulcan A−550).

Boehmite and alumina NPs were synthesized using the apparatus previously described under the conditions summarized in Table 1. The boehmite NPs were prepared using Al_2_(SO_4_)_3_·18H_2_O, p.a. (VWR chemicals, Radnor, PA, USA), dissolved in ultrapure water. The 24 mL of the prepared solution was placed in a plastic cuvette and mixed with ammonia (NH_3_, 25%, p.a., Gram mol, Zagreb, Croatia) was to tune the pH to 9. After mixing the reactants, white milky precipitates were obtained. The reaction mixture was placed in the microwave Teflon vessel and exposed to 200 °C for 30 min. The precipitates were centrifuged and washed with ultrapure water and ethanol and dried in a vacuum at 100 °C for 20 h. On the other hand, the alumina NPs were prepared by calcination at 800 °C for 4 h.

The synthesis of hematite NPs incorporated in different proportions into the MN1, MN2, and MN3 ion-selective membranes is described in detail in Ref. [25]. The magnetite NPs are synthesized by mixing iron(II) chloride tetrahydrate (FeCl_2_∙4H_2_O, p.a., 0.7952 g, VWR chemicals, Radnor, PA, USA), anhydrous iron(III) chloride (FeCl_3_, p.a., 8 mL, 1 mol L^−1^, Fluka, Charlotte, NC, USA), and ammonia (NH_3_, 25%, p.a., 5.4 mL, Gram mol, Zagreb, Croatia). The mixed solution was then transferred to a Teflon vessel and heated up to 200 °C for 10 min. After cooling, the precipitates were centrifuged and washed with ultrapure water and ethanol. The synthesis conditions are summarized in Table 2.

### 2.2. Ion-Selective Membranes

The main components of the ion-selective electrode presented in this work, i.e., ferric phosphate (FePO_4_), silver sulfide (Ag_2_S) and 4 different types of NPs, were preproduced or synthesized in our laboratory. The procedure for the preparation of FePO_4_, and Ag_2_S was described in detail in previous work [40]. The FePO_4_, Ag_2_S, Al, and Fe-based NPs, polytetrafluoroethylene (PTFE, p.a.) (Alfa Aesar, Haverhill, MA, USA) were weighted and pressed under 625 MPa for 2 h to form membranes weighing 500 mg with a 10 mm in diameter. Once the membrane was prepared, it was inserted into the electrode body and ultimately tested. The electrode body used in this work and represented in [40] was made of an epoxy plate with the cooper layer responsible for charge transfer between the membrane and cable connected to the millivoltmeter. Contact between the membrane and the copper layer was ensured with a conductive graphite adhesive. The copper layer was protected with a non-conductive varnish to avoid the influence of the testing solution. The composition of 12 different membranes, with indicated percentages for every component regarding total membrane mass, is shown in Table 3.

Membranes presented in Table 3 were tested in ferric nitrate nonahydrate [Fe(NO_3_)_3_·9H_2_O, p.a.), (VWR chemicals, Radnor, PA, USA), solution at pH of 1 and 5. The pH value 1 was adjusted with nitric acid (HNO_3_, p.a. Merck, KGaA, Darmstadt, Germany), while pH value 5 with acetic buffer prepared by mixing sodium acetate (CH_3_COONa, p.a., Kemika, Zagreb, Croatia) and acetic acid (CH_3_COOH, p.a., VWR chemicals, Radnor, PA, USA). As it was reported before, membranes with FePO_4_, Ag_2_S, and PTFE composition showed the best response at pH = 1 [40], and ferric selective electrodes modified with nanoparticles of iron oxides showed the best response at pH = 5 [20]. The tests were carried out at RT. Additionally, the possibility of quantitative application for some sensors was tested in standard solution (BDH chemicals, VWR 455532A iron standard solution for ICP, p.a., Radnor, PA, USA). The reference electrode used in potentiometric measurements was a double junction silver/silver chloride (Ag/AgCl) electrode (Reference plus, Mettler Toledo, Columbus, OH, USA). Both electrodes were immersed in the testing solution, which was positioned on a magnetic stirrer (Heildoph MR 300, Schwabach, Germany). The potential change was recorded by a millivoltmeter (SevenExcellence, Mettler Toledo, Columbus, OH, USA).

### 2.3. Characterization

The Al and Fe-based NPs were characterized by FTIR spectroscopy (Shimadzu IR Prestige−21, FTIR-8400S spectrophotometer, Kyoto, Japan) and PXRD measurements (Empyrean X-ray diffractometer with Cu Kα1 radiation, λ = 1.5406 Å, Malvern Panalytical Ltd. Malvern, Worcestershire, UK). Samples were scanned over a 2θ range between 10° and 75° with a scan step of 0.013°. Crystallite size information was extracted from the phase fitting method based on the change in profile widths compared to a standard sample. For insight into nanoparticles morphology, thermal field-emission scanning electron microscope ((FE-SEM), model JSM-7000F, manufactured by Jeol Ltd., Tokyo, Japan) was used, while the composition of the samples was checked with an energy dispersive spectrometer (EDS, model Oxford Inca 350, manufactured by Oxford Instruments, Abingdon, UK)

## 3. Results and Discussion

### 3.1. Bohemite NPs

**Figure 1:**
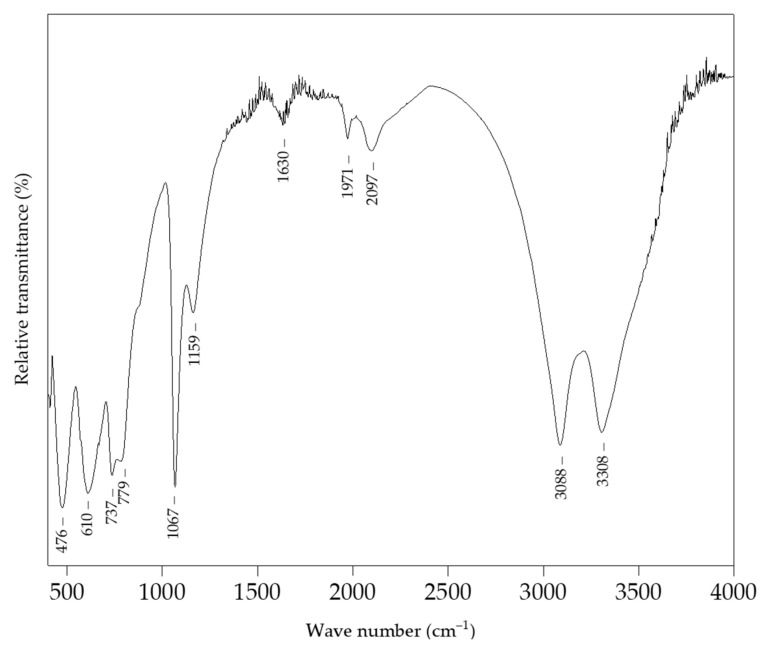
The FTIR spectrum of boehmite NPs.

The FTIR spectra of boehmite NPs is shown in Figure 1. The intense peaks located at 3308 and 3088 cm^−1^ originate from the O−H stretching, while the peak positioned at 1630 cm^−1^ indicates the presence of adsorbed water [41]. Peaks at 1067 cm^−1^ and 1159 cm^−1^ were characteristic for the symmetric and asymmetric Al−O−H bending, respectively, while the Al−O vibrations were located at 737, 610, and 476 cm^−1^ [42].

**Figure 2:**
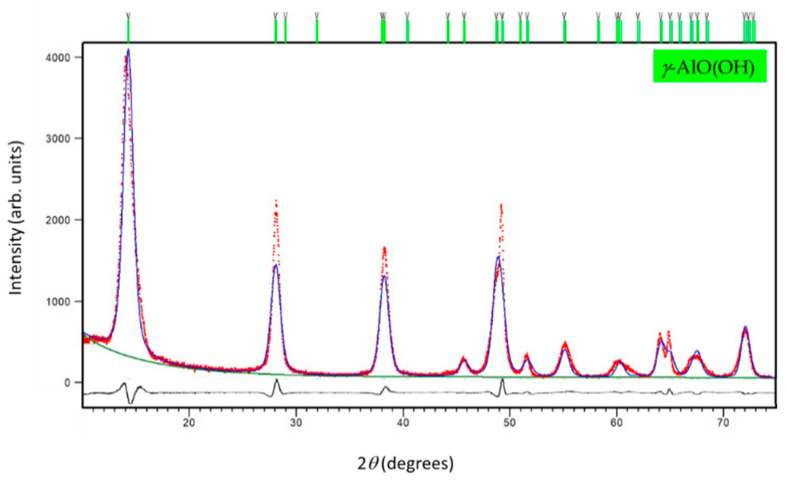
The final observed (red dots), calculated (blue solid line), and difference (black solid line) PXRD profiles of boehmite NPs at RT. The fitted background contribution is represented by the lower green solid line. The upper green tick marks show the reflection positions of the orthorhombic γ-AlO(OH) phase.

A single-phase PXRD pattern of boehmite preserves the crystal structure of orthorhombic γ-AlO(OH) exclusively with *Cmcm* space group [43] symmetry (*a* = 2.8612(6) Å, *b* = 12.244(7) Å, *c* = 3.6841(8) Å, and *V* = 129.06(8) Å^3^, *R*_wp_ = 7.31%) as indicated from the FTIR analysis and Rietveld refinement (see Figure 2). The value of the crystallite size obtained from the line-broadening analysis performed during the Rietveld structure refinements at RT was 11.7(1) nm.

**Figure 3:**
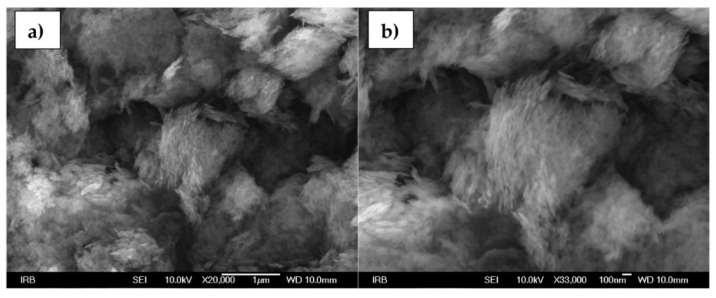
SEM images of boehmite NPs at (**a**) 20,000× and (**b**) 33,000× magnification.

Figure 3a,b shows SEM images of boehmite NPs. Magnifications from 20,000× up to 33000× are represented and nanosized, (<20 nm in diameter), needle-shaped particles are visible under higher magnifications [44,45].

**Figure 4:**
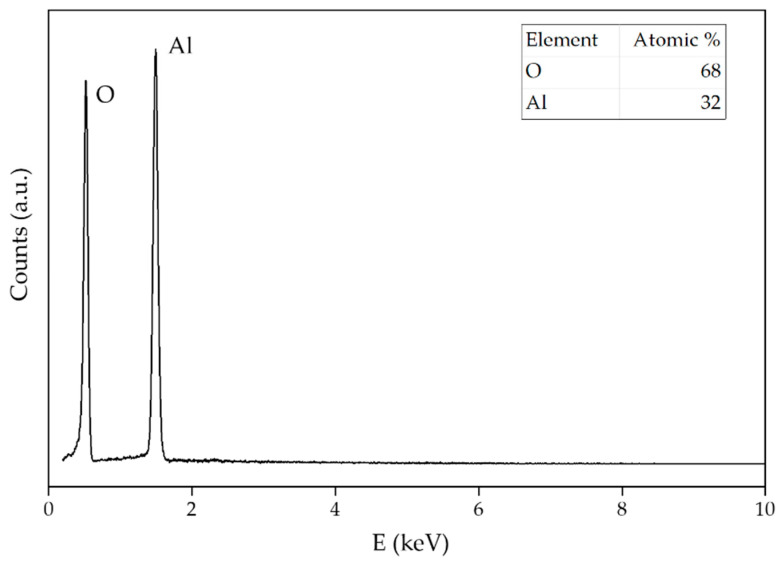
The EDS pattern of boehmite NPs. The results of the element analysis are shown in the inset.

Figure 4 represents EDS data of boehmite NPs. The atomic ratio of 32% Al and 68% O matches for γ-AlO(OH) particles.

### 3.2. Alumina NPs.

**Figure 5:**
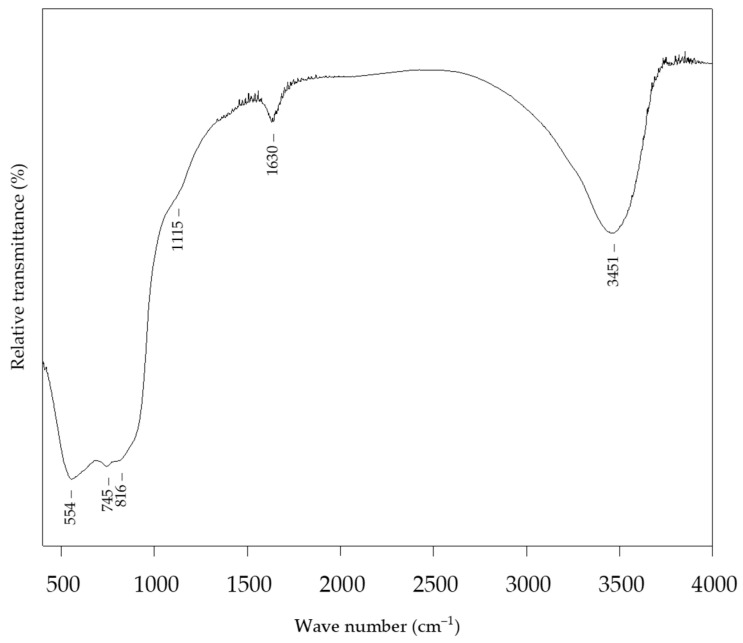
FTIR spectrum of alumina NPs.

The FTIR spectra of the alumina NPs (Figure 5) show the presence of adsorbed water, as indicated by peaks located at 3451 cm^−1^ and 1630 cm^−1^. The characteristic peak at 1115 cm^−1^ can be attributed to the Al−O bond stretching. The peaks at 745 cm^−1^ and 554 cm^−1^ were attributed to the symmetric stretching and bending vibrations of the Al−O−Al bond, respectively [46].

**Figure 6:**
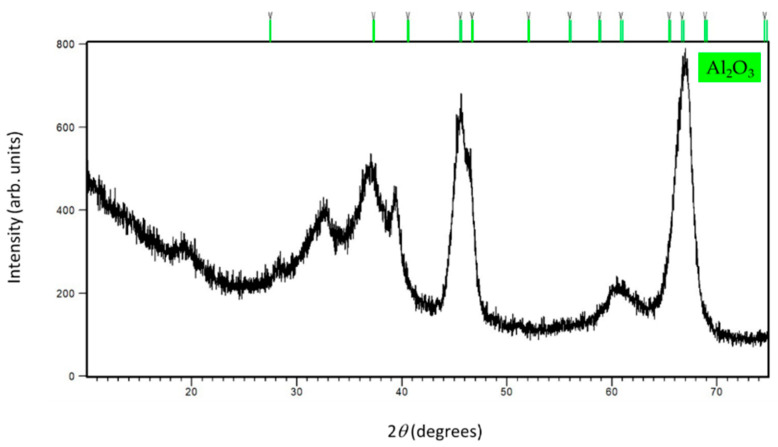
The final observed PXRD profile of alumina NPs at RT. The upper green tick marks show the reflection positions of the hexagonal Al_2_O_3_ phase formation.

The PXRD pattern (Figure 6) of the alumina sample shows an indication of the hexagonal Al_2_O_3_ formation. Undoubtedly, X-ray diffraction indicates the appearance of an amorphous phase and a rather poor crystallinity of this sample.

**Figure 7:**
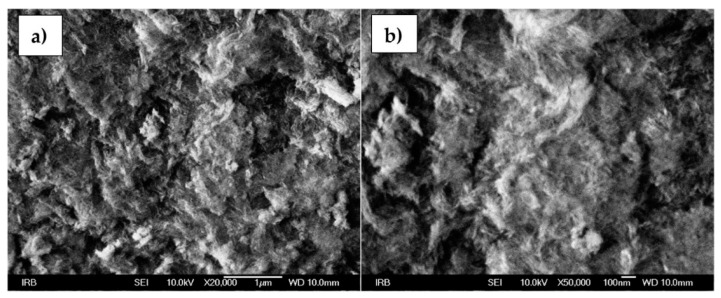
SEM images of alumina NPs at (**a**) 20,000× and (**b**) 50,000× magnification.

The morphology of alumina NPs is shown in Figure 7a,b. Under a high magnification of 50,000×, as with the boehmite NPs, a needle-like structure is visible for the alumina NPs as well.

**Figure 8:**
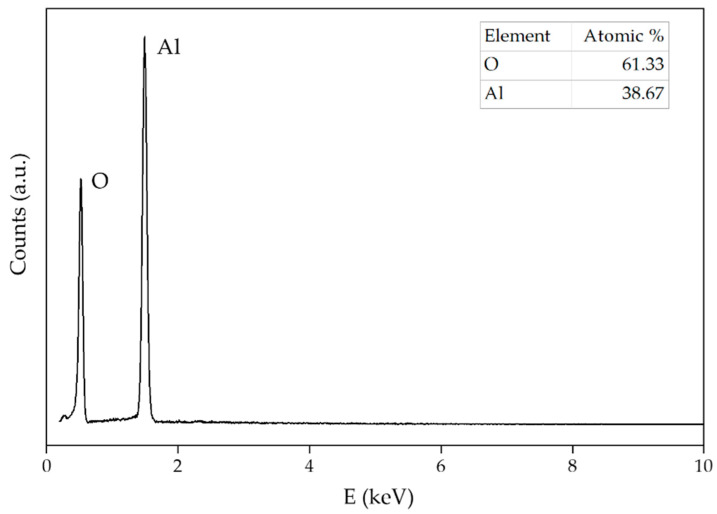
EDS pattern of alumina NPs. The results of the element analysis are shown in the inset.

The EDS spectrum of alumina NPs (Figure 8) shows a stoichiometric Al/O ratio almost ideal for alumina. The absence of any other element besides aluminum and oxygen proves sample purity.

### 3.3. Magnetite NPs

**Figure 9:**
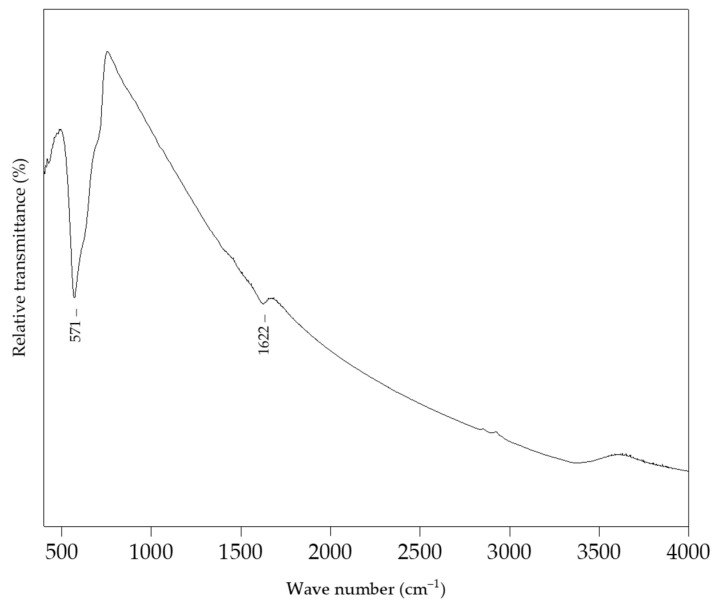
FTIR spectrum of magnetite NPs.

The FTIR spectrum of magnetite NPs is shown in Figure 9, indicating a characteristic Fe−O stretching vibration located at 571 cm^−1^ [47].

**Figure 10:**
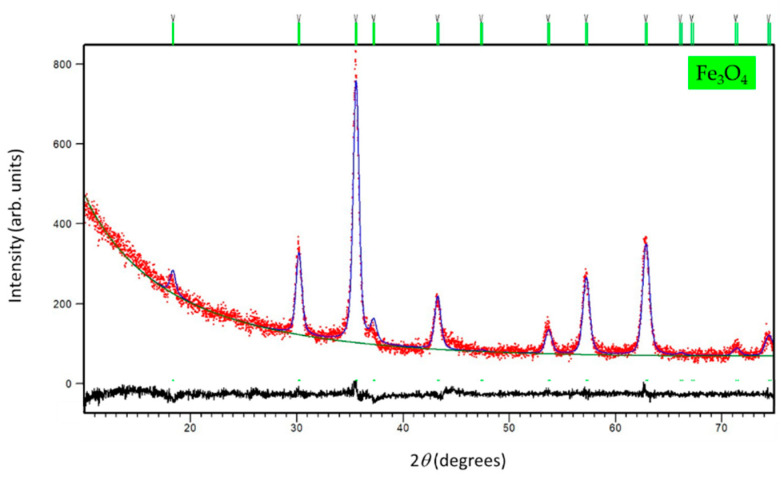
The final observed (red dots), calculated (blue solid line) and difference (black solid line) PXRD profiles of magnetite NPs at RT. The fitted background contribution is represented by the lower green solid line. The upper green tick marks show the reflection positions of the cubic Fe_3_O_4_ phase.

The PXRD pattern of magnetite NPs confirmed a formation of the phase pure sample. As obtained from the Rietveld structure refinement at RT (see Figure 10), the unit cell metrics was assigned to cubic Fe_3_O_4_ symmetry [48] (s. g. *Fd*-3*m*, a = 8.357(1) Å, *V* = 583.56 Å3, *R*_wp_ = 5.32%). The crystallites of 14.2(1) nm were calculated to form the line-broadening analysis.

**Figure 11:**
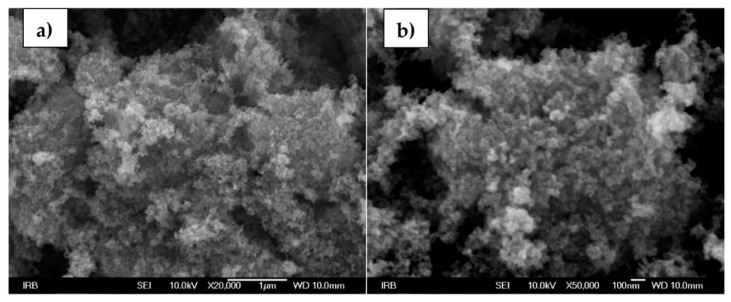
SEM images of magnetite NPs at a (**a**) 20,000× and (**b**) 50,000× magnification.

Figure 11a,b shows SEM images of magnetite NPs. Irregularly shaped aggregates are visible on SEM micrographs of magnetite. The individual magnetite nanoparticles within the aggregates are smaller than 20 nm.

**Figure 12:**
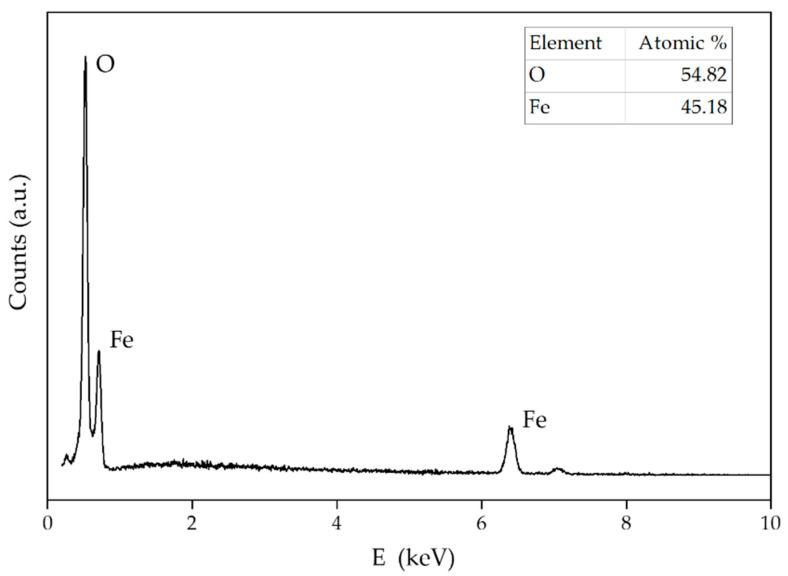
EDS pattern of magnetite NPs. The results of the element analysis are shown in the inset.

The EDS spectra of magnetite NPs (Figure 12), apart from the ideal ratio of iron and oxygen for this type of NPs, do not show the presence of any impurities. The atomic ratio for magnetite NPs was 54.82% of oxygen and 45.18% of iron.

Hematite NPs complete characterization is reported in Ref. [25].

### 3.4. Membranes with Alumina, Boehmite, Magnetite and Hematite NPs

The response of a solid-state ion-selective electrodes is generally based on ion exchange processes occurred between the solution phase and the solid phase of the sensor. Since the membranes presented in this paper contain iron(III) phosphate, the reaction that takes place on the surface of the membrane is:

(1) $${\mathrm{Fe}3^{+}}_{(\mathrm{aq})(\mathrm{solution})\;}+{{\mathrm{PO}}_{4}3^{-}}_{\left(\mathrm{aq})(\mathrm{membrane}\right)}⇄\;{\mathrm{Fe}({\mathrm{PO}}_{4})}_{\left(s\right)}$$

**Figure 13:**
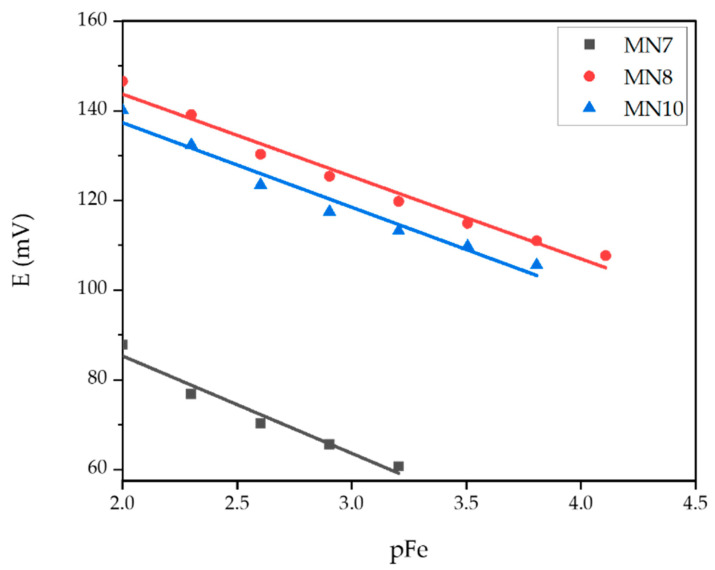
Potential change dependence of pFe evaluated for MN7, MN8, and MN10 membranes.

Table 4 summarizes the most important features of membranes with the addition of aluminum oxide and boehmite NPs in different percentages according to total membrane mass. Results obtained for MN7 and MN8 membranes are from tests under strongly acidic conditions (pH = 1), while results for MN10 were obtained under weakly acidic conditions (pH = 5). The calibration curves for MN7, MN8, and MN10 membranes are represented in Figure 13.

The MN7 membrane with an addition of 0.25% alumina NPs showed a linear response to ferric cations in Fe(NO_3_)_3_ solution at pH 1. The recorded slope was −21.73 mV per decade with a correlation factor of 0.9635. The addition of a larger amount of alumina NPs in the MN8 membrane (0.5%) had a positive effect on extending the linearity range of the membrane. Accordingly, the linearity of this membrane was measured in a concentration range from 7.81·10^−5^ mol L^−1^ to 1·10^−2^ mol L^−1^. The slope obtained in the mentioned concentration range was −18.75 mV per decade with a correlation factor of 0.9774. Therefore, it is obvious that the increased content of alumina NPs had a positive effect on the membrane properties, considering the increase of the linear dynamic range for the whole decade as well as the sensitivity. The membranes MN7 and MN8 did not show significant results at pH 5. On the other hand, the MN10 membrane with an addition of 0.25% boehmite NPs showed linearity in the determination of ferric cations in the concentration range of 1.56·10^−4^–1·10^−2^ mol L^−1^ with a slope of −18.86 mV per decade and a correlation factor of 0.9674, while tested in acetic buffer at pH 5. The detection limit for this membrane was 8.38·10^−5^ mol L^−1^. Since the ideal slope for the determination of trivalent cations according to the Nernst equation was −19.73 mV per decade, the slopes obtained with the membranes MN7, MN8, and MN10 agreed quite well with the theoretical requirements.

**Figure 14:**
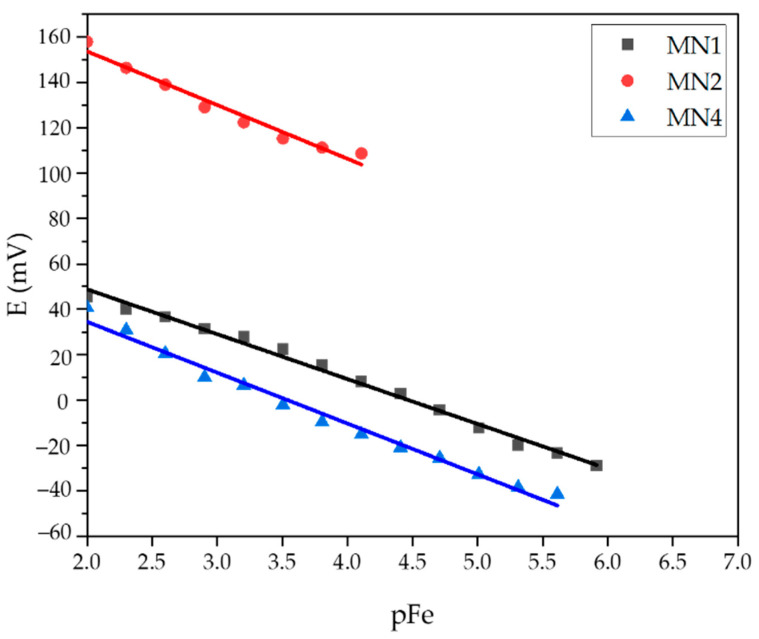
Potential change dependence of pFe evaluated for MN1, MN2, and MN4 membranes.

A summary of test results for membranes containing iron oxide NPs is given in Table 5. The membranes were tested in a precisely prepared Fe(NO_3_)_3_ ·9H_2_O solution at pH 1 and 5. The calibration curves for MN1, MN2 (pH = 5), and MN4 (pH = 1) membranes are shown in Figure 14.

The MN4 membrane with 0.25% magnetite NPs showed linearity in the determination of iron cations in the concentration range of 2.44·10^−6^ to 10^−2^ mol L^−1^ with a slope of −22.38 mV per concentration decade and a correlation factor of 0.9853. The detection limit for this membrane was 1.85·10^−6^ mol L^−1^. The MN1 and MN2 membranes contained 0.25% and 0.50% hematite NPs, respectively. The MN2 membrane, with 0.50% hematite showed −23.64 mV per decade slope in the range from 7.81 × 10^−5^ to 10^−2^ mol L^−1^ of ferric cations concentration.

**Figure 15:**
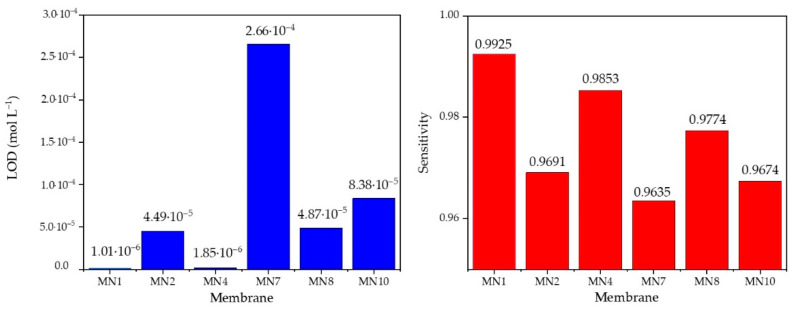
A comparative bar graph for LOD and sesnsitivity values fo MN1, MN2, MN4, MN7, MN8, and MN10 membranes.

To obtain a better insight into the differences in the values of the limit of determination and the sensitivity between the presented membranes, the results from the tables above are also presented in the comparative bar graph (Figure 15).

When analyzing the data from Table 5, the addition of 0.25% hematite had the greatest effect on the extension of the linear dynamic range of the membrane as well as on the detection limit, which plays an important role in this measurement method. As mentioned before, an ideal theoretical slope for trivalent cations determination was established using the Nernst equation and was −19.3 mV per one concentration decade. Since MN1 membrane has a slope of −19,75 mV per decade, it is obvious that it is an ideal one. The correlation factor was also very close to ideal value (~1) as *R*^2^ = 0.9925. The detection limit of 1.01·10^−6^ was approximately the lower limit of the potentiometry method. Since the MN1 sensor responded best to the determination of ferric cations, this membrane was verified by the control experiments for the quantitative determination of ferric cations. Table 6 shows the results of the test of MN1 membrane in iron standard (VWR 455532A, BDH chemicals, plasma emission standard) at pH 5 with the calculated values using the calibration curve *E* = −19.753pFe + 88.334 mV obtained just before the test in the standard solution.

The measurements for the same concentration of ferric cations are repeated three times intraday, and the arithmetic middle of these measurements showed a rather high recovery value of 99.4%

**Figure 16:**
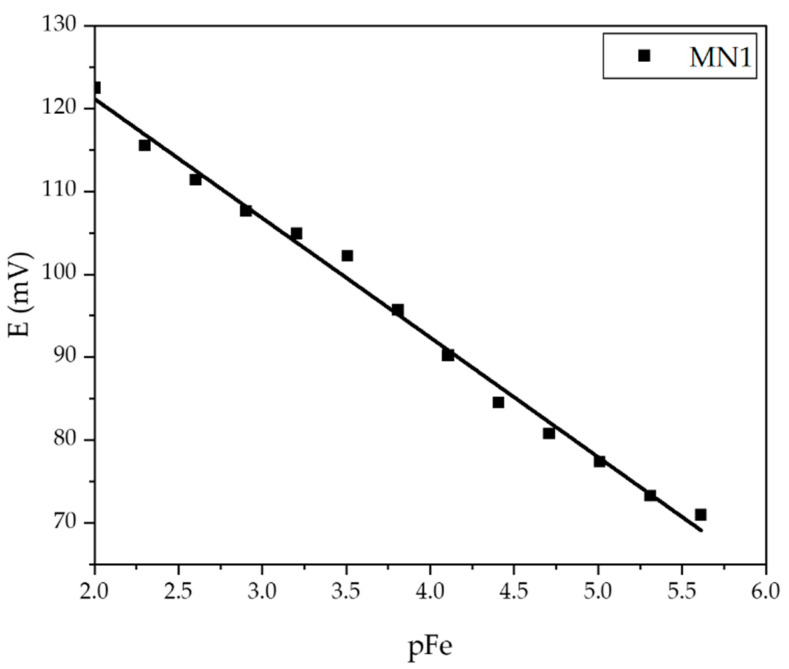
Potential change dependence of pFe evaluated for MN1 membrane in standard solution.

After testing in a laboratory prepared Fe(NO_3_)_3_ solution, the MN1 membrane was tested in an iron standard solution using sequential dilution method and obtained calibration curve (Figure 16) was used to verify the possibility of a quantitative application of the proposed membrane (Table 7).

The slope recorded during MN1 testing in iron standard solution was −14.405 mV per decade with 0.9931 correlation factor. The linear range was 2.44 × 10^−6^−10^−2^ mol L^−1^ ferric cations concentration.

The high arithmetic recovery values listed in Table 7 confirm the possibility of using the MN1 sensor for the quantitative determination of ferric cations. The lowest recovery value (among arithmetic values of three measurements), obtained for 1.1169 mg of analyte in solution was 93.6%. However, it is also important to consider the reproducibility values in the context of the agreement of the potential of the calculated and measured values. Evidently, the difference of only 0.4 mV between calculated and measured potential change causes 6.4% uncertainty if analyte masses are compared in solution. Thus, there is a big difference in results interpretation according to ion charge number, and this must be taken into account when considering the effectiveness of a particular sensor. This sensor showed a limited lifetime of approximately one month. During testing processes, the changes in potential as a function of the changing concentration of ferric cations and reading times of potential were recorded. The approximate time for reading was 30 s.

### 3.5. Surface of an Ion-Selective Membrane

**Figure 17:**
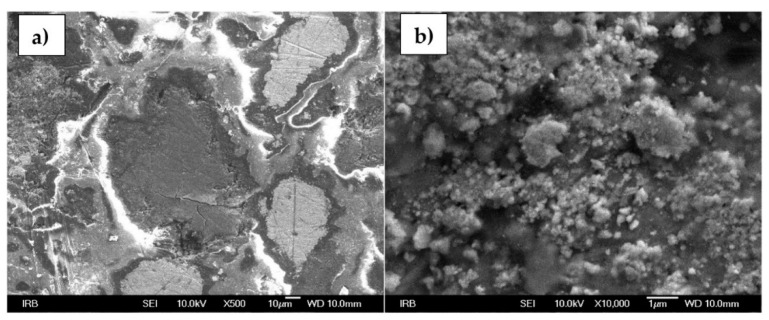
SEM images of MN1 membrane surface at a (**a**) 500× and (**b**) 10,000× magnification.

Figure 17a,b represents the morphology of the tested MN1 sensor. At 500× magnification, a very heterogeneous surface is noticeable, unlike one that could be seen by an eye. Lines visible under 500× magnification are the result of membrane polishing. In addition, the PTFE, since it is an insulator, interfered with the resolution quality when recording large magnifications. Different sizes and particle agglomerations are noticeable at 10,000× magnification.

## 4. Conclusions

Boehmite and alumina NPs as well as magnetite and hematite, were synthesized under very precise conditions and characterized by FTIR and PXRD followed by Rietveld analysis to determine the structural and microstructural features. Additionally, the samples were characterized by FE-SEM and EDS to determine the shape of the particles. Not only detailed synthesis and characterization of NPs was represented but also the possibility of their application in the modification of the ion-selective electrode. As reported in previous work, membranes with only three main components; FePO_4_, Ag_2_S, and PTFE showed a smaller linear range for ferric cations determination in regard to membranes enriched with specific NPs types. Electrodes with an addition of alumina and boehmite NPs showed less desirable results in comparison to the results obtained when testing sensors were modified with hematite and magnetite NPs. The results of testing the MN1 membrane having composition FePO_4_:Ag_2_S:PTFE = 1:1:2 with the addition of 0.25% hematite NPs, particularly stand out. The slope of −19.75 mV per decade and 0.9925 correlation factor are in almost ideal agreement with the requirements of the Nernstian equation for the ion-selective electrodes for trivalent cations. The detection limit of 1.01·10^−6^ mol L^−1^ is very close to the lower detection limit of ion-selective electrodes. Recovery values for ferric cations determination were 99.4% for the membrane when calibration curve was performed in Fe(NO_3_) 9H_2_O solution and 106.7%, 93.6%, and 101.1% when calibration curve was performed in standard iron solution. Such high values prove the possibility of quantitative determination of analytes in a wide range of concentrations. The lifetime of sensors was approximately one month with only 30 s of detection time. This way, a new homemade membrane for an ion-selective electrode was constructed and presented.

## Figures and Tables

**Table 1:** Experimental conditions for boehmite and alumina NPs preparation.

Sample	0.1 M Al_2_(SO_4_)_3_∙18H_2_O (mL)	25% NH_3_ (mL)	Temperature (°C)	Time (min)	Drying Temperature (°C)	Calcination Temperature (°C)	pH
boehmite	24	~3.7	200	30	100	−	9
alumina	24	~3.7	200	30	100	800	9

**Table 2:** Experimental conditions for magnetite NPs preparation.

Sample	1 M FeCl_3_ (mL)	25% NH_3_ (mL)	FeCl_2_·4H_2_O (g)	Temperature (°C)	Time (min)	Drying Temperature (°C)	pH
magnetite	8	~5.4	0.792	200	10	60	8.7

**Table 3:** Composition of the membranes for the determination of the ferric cation.

Membrane	Membrane Mixture Composition (%)						
	FePO_4_	Ag_2_S	PTFE	NP Type			
				Hematite	Magnetite	Alumina	Boehmite
MN1	25	25	50	0.25			
MN2	25	25	50	0.5			
MN3	25	25	50	1			
MN4	25	25	50		0.25		
MN5	25	25	50		0.5		
MN6	25	25	50		1		
MN7	25	25	50			0.25	
MN8	25	25	50			0.5	
MN9	25	25	50			1	
MN10	25	25	50				0.25
MN11	25	25	50				0.5
MN12	25	25	50				1

**Table 4:** Test results using membranes modified with boehmite and alumina NPs.

Membrane	Slope (mV dec^−1^)	Linear Range (mol L^−1^)	LOD (mol L^−1^)	*R* ^2^
MN7	−21.73	6.25·10^−4^–1·10^−2^	2.66·10^−4^	0.9635
MN8	−18.37	7.81·10^−5^–1·10^−2^	4.87·10^−5^	0.9774
MN10	−18.86	1.56·10^−4^–1·10^−2^	8.38·10^−5^	0.9674

**Table 5:** Test results using membranes modified with magnetite and hematite NPs.

Membrane	Slope (mV dec^−1^)	Linear Range (mol L^−1^)	LOD (mol L^−1^)	*R* ^2^
MN4	−22.38	2.44·10^−6^–1·10^−2^	1.85·10^−6^	0.9853
MN1	−19.75	1.22·10^−6^–1·10^−2^	1.01·10^−6^	0.9925
MN2	−23,64	7.81·10^−5^–1·10^−2^	4.49·10^−5^	0.9691

**Table 6:** Test results for MN1 in standard solution for *m*(Fe^3+^) = 1.1169 mg.

	*m*(Fe^3+^) = 1.1169 mg; *E*_calc._ = 23.1 mV			
	1st Cycle	2nd Cycle	3rd Cycle	Average
*E*_(measured)_ (mV)	23.3	23.1	22.8	23.07
*m*(Fe^3+^)_measured_ (mg)	1.1394	1.1169	1.0749	1.1104
recovery (%)	102.01	100	96.2	99.4

**Table 7:** Test results for MN1 in standard solution for *m*(Fe^3+^) = 2.238 mg, *m*(Fe^3+^) = 1.1169 mg and *m*(Fe^3+^) = 0.2234 mg.

	*m*(Fe^3+^) = 2.2338 mg; *E*_calc_ = 106.8 mV			
	1st Cycle	2nd Cyle	3rd Cycle	Average
*E*_measured_ (mV)	108	107	106.4	107.1
*m*(Fe^3+^)_measured_ (mg)	2.7213	2.3193	2.1072	2.3826
recovery (%)	121.8	103.8	94.3	106.7
	*m*(Fe^3+^) = 1.1169 mg; *E*_calc_. = 102.4 mV			
	1st cycle	2nd cycle	3rd cycle	average
*E*_measured_ (mV)	102.5	102	101.5	102
*m*(Fe^3+^)_measured_ (mg)	1.1297	1.0429	0.9628	1.0451
recovery (%)	101.1	93.4	86.2	93.6
	*m*(Fe^3+^) = 0.2234 mg; *E*_calc_. = 92.4 mV			
	1st cycle	2nd cycle	3rd cycle	average
*E*_measured_ (mV)	92.9	92	92.4	92.4
*m*(Fe^3+^)_measured_ (mg)	0.2435	0.2109	0.2234	0.2259
recovery (%)	109.0	94.4	100	101.1

## Data Availability

Not applicable.
